# Sound Insulation in a Hollow Pipe with Subwavelength Thickness

**DOI:** 10.1038/srep44106

**Published:** 2017-03-08

**Authors:** Hai-Long Zhang, Yi-Fan Zhu, Bin Liang, Jing Yang, Jun Yang, Jian-Chun Cheng

**Affiliations:** 1 Key Laboratory of Modern Acoustics, MOE, Institute of Acoustics, Department of Physics, Nanjing University, Nanjing 210093, P. R. China; 2 Collaborative Innovation Center of Advanced Microstructures, Nanjing University, Nanjing 210093, P. R. China; 3 Key Laboratory of Noise and Vibration Research, Institute of Acoustics, Chinese Academy of Sciences, Beijing 100190, P. R. China

## Abstract

Suppression of the transmission of undesired sound in ducts is a fundamental issue with wide applications in a great variety of scenarios. Yet the conventional ways of duct noise control have to rely on mismatched impedance or viscous dissipation, leading the ducts to have ventilation capability weakened by inserted absorbers or a thick shell to accommodate bulky resonators. Here we present a mechanism for insulating sound transmission in a hollow pipe with subwavelength thickness, by directly reversing its propagating direction via anomalous reflection at the flat inner boundary with well-designed phase profile. A metamaterial-based implementation is demonstrated both in simulation and in experiment, verifying the theoretical prediction on high-efficient sound insulation at the desired frequencies by the resulting device, which has a shell as thin as 1/8 wavelength and an entirely open passage that maintains the continuity of the background medium. We have also investigated the potential of our scheme to work in broadband by simply cascading different metamaterial unit cells. Without the defects of blocked path and bulky size of existing sound insulators, we envision our design will open new route to sound insulation in ducts and have deep implication in practical applications such as designs of ventilation fans and vehicle silencers.

Elimination of the transmission of undesired noise is of fundamental significance and potential application in the field of acoustics^1^,^2^,^3^. Among the numerous applications of sound insulation, duct noise control^4^,^5^,^6^ is of particular interests from both physics and engineering points of view. The past several decades have witnessed considerable efforts dedicated to the development of both active and passive methods for noise control. The methods of active noise control^7^ need to introduce another sound source with antiphase, while passive methods^1^,^2^,^3^,^4^,^5^,^6^ provide a simple and low cost solution requiring no power supply. Conventionally, the sound insulation in ducts has to rely on reflection effect by mismatched impedance such as with the expansion-chamber-type muffler or by viscous dissipation such as with fibrous duct lining in air distribution systems^5^. However, the insertion of porous media in the sound path obviously impair the crucial ventilation function of the ducts and, on the other hand, the usage of Helmholtz resonators^8^,^9^,^10^,^11^ or membranes^4^,^6^ may make them impractical for many important applications, esp., sound insulation in hollow and thin-shelled pipes with no space for decorating resonators with bulky size under its inner surfaces. It is therefore of both physical and practical significance to explore new mechanisms producing sound insulation to break through these limitations in conventional methods.

In this article, we propose to insulate acoustic transmission in hollow pipes by directly manipulating the propagation direction with specially-engineered phase profile at the inner boundary, fundamentally different from the traditional methods that depend on either impedance mismatch or viscous effect. This should be a universal mechanism that applies to ducts with different geometries, as long as the profile of phase delay on the inner boundary can be controlled as desired. The desired phase profile is produced by employing the recently-emerged metasurfaces^12^,^13^,^14^,^15^,^16^ which have potentials to achieve novel phenomena such as extraordinary refraction/reflection^17^,^18^,^19^, beam forming^20^,^21^,^22^,^23^, asymmetric transmission^24^,^25^, and vortices generation^26^. An acoustic metasurface (AM) based implementation of our proposed scheme is demonstrated both numerically and experimentally. Good agreement is observed between the numerical results and the experimental data, with both showing that the designed insulator effectively blocks the transmission of incident wave within the prescribed frequency range despite its flat shape and open configuration. We anticipate our finding to constitute a significant step beyond the existing duct noise control methods that generally suffers from blocked sound path and bulky size, therefore bringing promising applications in a great variety of practical scenarios ranging from ventilation fans to vehicle silencers with need for open passage.

## Results

**Figure 1:**
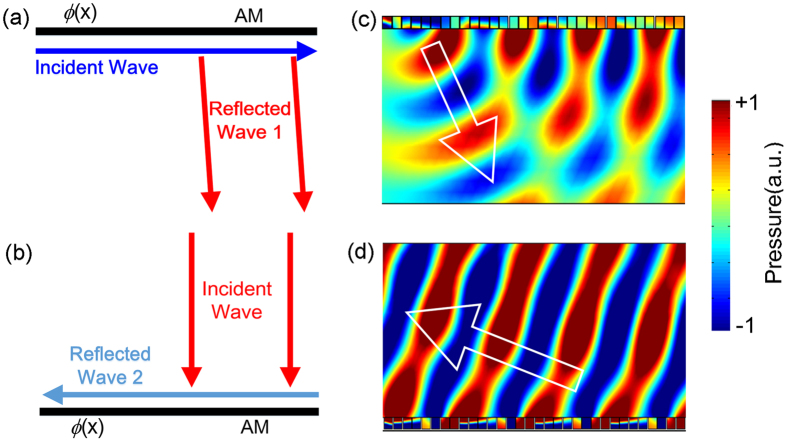
Schematic illustration of sound insulation by manipulating the wave vector of incident wave. (**a**) The anomalous reflection on the upper boundary that bends the propagation direction of grazing-incidence wave by approximately*π*/2. (**b**) The anomalous reflection on the bottom boundary that eventually reverses the direction of incident wave. (**c**,**d**) Scattered sound pressure field of the anomalous reflection for (**c**) grazing incidence and (**d**) vertical incidence in the condition of effective parameters model. Propagation direction of scattered wave is marked by white arrows.

The basic idea of our proposed scheme is schematically shown in Fig. 1, which aims to bounce back the incident wave in an intuitive manner. For simplicity without losing generality, we consider a two dimensional (2D) case. Given that the proposed structure needs to keep open and contains no layered materials in the propagating path, one has to rely on the interaction between the incident wave and the flat inner surfaces of the pipe. In this context, the most straightforward way to reflect back the incident wave might be bending its direction of propagation twice to form a U-shape trajectory as shown in Fig. 1(a) and (b) which display the desired trajectory for the wave incident in the upper part of the pipe, due to the incapability of a flat surfaces to directly block the transmission of wave travelling across it. However such a design apparently needs anomalous reflection at the inner boundaries on both sides, which goes beyond the conventional concept of equivalent incident and reflected angles and has to be governed by the generalized Snell’s law which is deduced by Fermat’s principle^27^,^28^,^29^. By engineering the microstructure on flat surface to produce appropriate phase gradient, the direction of reflected wave can be predicted by 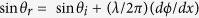, where *θ*_*r*_(*θ*_*i*_) is the reflected (incident) angle, *λ* is the wavelength of sound wave and *dϕ*/*dx* is the phase gradient. Note that in our design the propagation direction of incident wave needs to be bent by approximately *π*/2 when travelling along the two boundaries. By making the phase gradient term equal to −*k* with *k* being the wave vector of incident wave, the reflected angle can be controlled to be nearly zero, leading the reflected wave to impinge on the opposite boundary normally. On the opposite surface with the same phase profile, the reflected wave would be bent again to propagate along a direction almost reverse to the original incidence direction due to the symmetry of the structure, preventing the incident acoustic energy from passing through the system. Such theoretical analysis predicts that by properly modulating the reflection phase profile on the inner surface of its shell, regardless of the thickness of the shell itself, the proposed structure would have the potential to yield high-efficient sound insulation. Typical numerical results in Fig. 1(c) and (d) show respectively the scattered sound pressure fields yielded by the two opposite boundaries with the aforementioned phase profiles, which are mimicked by two arrays of unit cells characterized by ideal effective acoustical parameters (marked by black boxes in the figures). Here the phase distribution along the two boundaries is discretized for facilitating the experimental realization which will be demonstrated later. The results verify the occurrence of the expected anomalous reflections on the two boundaries that eventually bend the wave vector of the incident wave approximately to the reverse of the original direction. This suggests the potential of the combination of the two boundaries to serve as an effective sound insulator for blocking the incident wave, as will be proven both theoretically and experimentally later.

**Figure 2:**
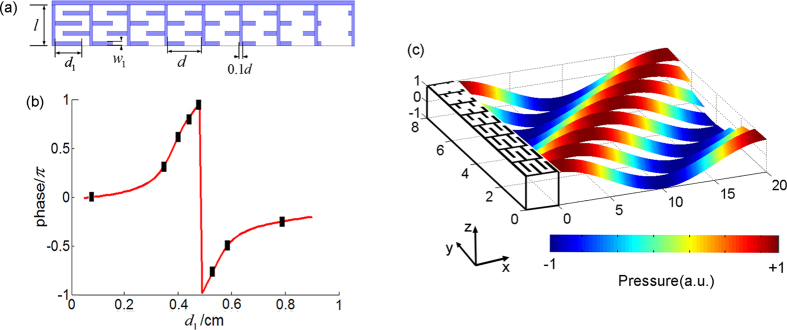
An AM by coiling up space for generalized Snell’s law. (**a**) One period of the proposed metasurface with eight units and its structural parameters. (**b**) Dependence of the reflected phase on the width *d*_1_ marked by black dots for an individual unit cell of labyrinthine metastructure contained in the structure shown in (**a**). (**c**) The pressure stripes of the acoustic waves which are reflected by the eight units. The high maps of pressure field are utilized to clearly show the different phase shifts by each unit at working frequency of 4346 Hz.

In what follows we will demonstrate a practical implementation of our scheme by employing the recently-emerged AMs capable of providing discrete phase with thickness much smaller than the working wavelength. A basic building block of the designed acoustic metasurface is shown in Fig. 2(a), which is constructed by four identical thin rigid plates in air (length *d*_1_ and width *w*_1_), leaving the space with a width of *w*_2_ between the plates as a channel for effectively delaying the propagation of sound inside it^30^. It should be noted that, since acoustic waves, as scalar waves, can propagate within the channels freely, the delayed phase of reflected waves on the metasurface can be retrieved. Dependence of reflected wave phase on the width *d*_1_ is plotted in Fig. 2(b) with red solid line. Despite the subwavelength scale of the overall thickness of such a labyrinthine unit which is chosen as *l *= 0.128*λ* in this study, a sufficient phase shift can be achieved by coiling up space. In our design, the structural parameters are chosen as *w*_1_ = 0.1 cm, *l *= 1 cm and *d* = 1 cm. Through carefully selecting the values of *d*_1_ for the eight units to cover the 2*π* span with a discrete phase step of *π*/4, as marked by the eight black dots in Fig. 2(b), the desired phase gradient can be produced on the metasurface. To further verify the ability of the designed AM to produce discrete phase shifts, the reflected waves by these eight units are shown in Fig. 2(c). The stripes refer to the pressure filed patterns at the same time instant. Here the *x* axis represents the reflected phase delayed by the eight different unit cells which are arranged along the *y* axis while the *z* axis is the meaning of pressure amplitude. The peak of the pressure field can shift up to a wavelength, making it possible to give rise to the desired discrete phase shifts by the eight units.

**Figure 3:**
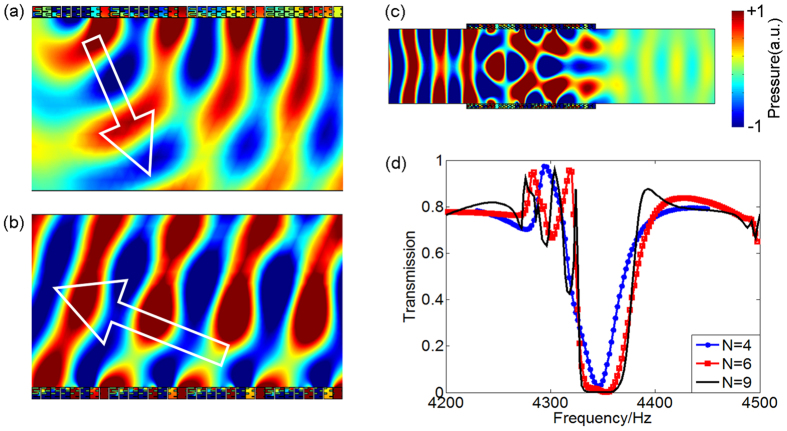
AM with coiled-up space for composing the sound insulator. Distribution of acoustic pressure of the scattered wave field produced by the anomalous reflection on the AM for (**a**) grazing incidence and (**b**) normal incidence cases. Propagation direction of scattered wave is marked by white arrows and the incident wave is not shown in the figures. (**c**) Simulated distribution of pressure amplitude in a model with four periods (*N* = 4) of unit cells on each side at 4346 Hz. (**d**) Comparison between the frequency dependences of the sound intensity transmission for three cases with different periods, i.e., *N* = 4, 6 and 9.

Then we numerically calculated the distribution of acoustic pressure of the scattered wave generated by an AM implemented by the above-mentioned labyrinthine metastructures, whose structural parameters are well tuned so as to yield specific phase profiles shown in Fig. 1. Simulated results for two cases of grazing incidence and normal incidence, shown in Fig. 3(a) and (b) respectively, are almost identical with the results obtained for an ideal model displayed in Fig. 1, showing that the metamaterial-based AMs produce anomalous reflection exactly along the predicted direction. As a result, by arranging such unit cells periodically in a row to realize the required phase distribution, we form a hollow pipe capable to reflect back most of the incident acoustic energy as expected. Figure 3(c) demonstrates the simulated acoustic pressure field in the pipe at the frequency of 4346 Hz. A comparison between the transmissions as functions of frequency for systems with different periods is shown in Fig. 3(d), showing the possibility to further improve the performance of the insulator through simply increasing the period numbers, but at the cost of enlarging the length of the whole structure, as will be demonstrated later.

**Figure 4:**
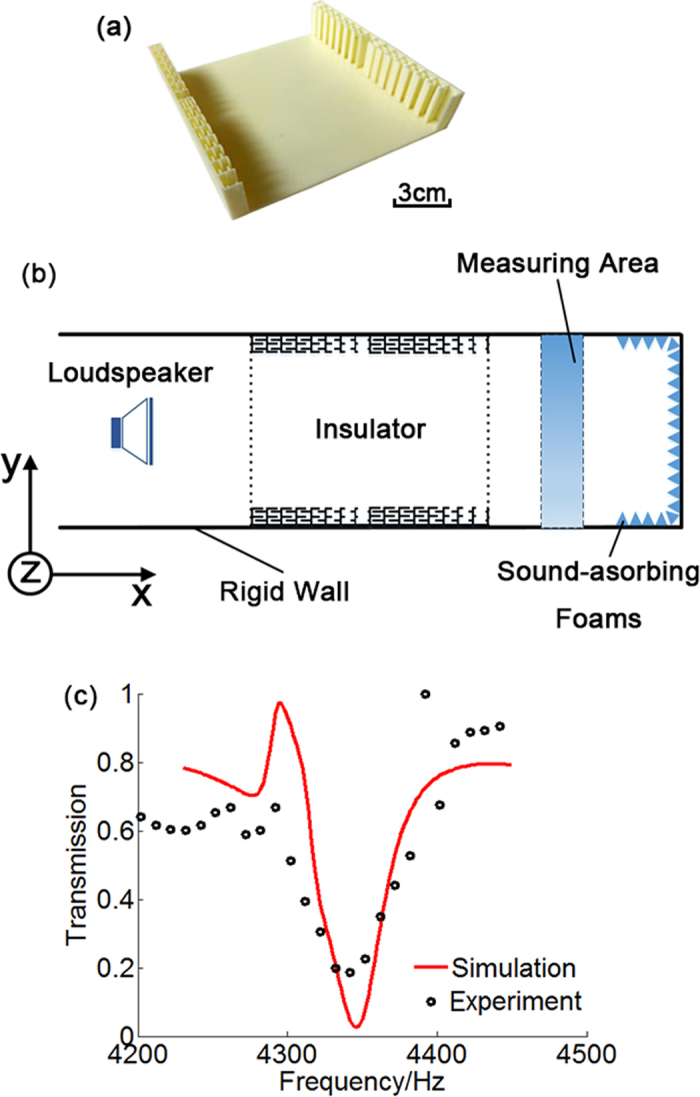
Experimental setup and measured results. (**a**) Photograph of the proposed model with length 17.6 cm in the *x* direction and width 17 cm in the *y* direction. (**b**) Schematic of experimental setup. (**c**) Transmission coefficient of simulation and measured results in the case of four unit cells at working frequency of 4346 Hz.

Experiments are designed and carried out to verify the realization of sound insulation in the proposed hollow pipe with shells much thinner than the operating wavelength. A sample with four periods of labyrinthine units on each side is fabricated with acrylonitrile-butadiene-styrene (ABS) plastic via three dimensional (3D) printing technique (Stratasys Dimension Elite, 0.177 mm in precision) as shown in Fig. 4(a). In both the simulation and experiment, the transmissions are obtained by integrating the sound power along the cross-section of the waveguide structure. Figure 4(c) displays the experimental results of transmission coefficients of the proposed hollow pipe. Numerical simulations are also performed for a quantitative comparison, with corresponding results shown in Fig. 4(c) as well. Good agreements are observed between the simulated and measured results, with both demonstrating that the propagation of incident wave in the fabricated sample is virtually blocked with the maximal reduction in transmission coefficients achieved at the designed frequency 4346 Hz. The slight discrepancy should come from the imperfect sample fabrication and non-zero reflection at the end. As a consequence, the proposed scheme is verified effective to insulate sound at the desired frequency with sound path totally open to other entities like flows or lights and with no need for bulky-sized resonators decorated under the inner surfaces.

**Figure 5:**
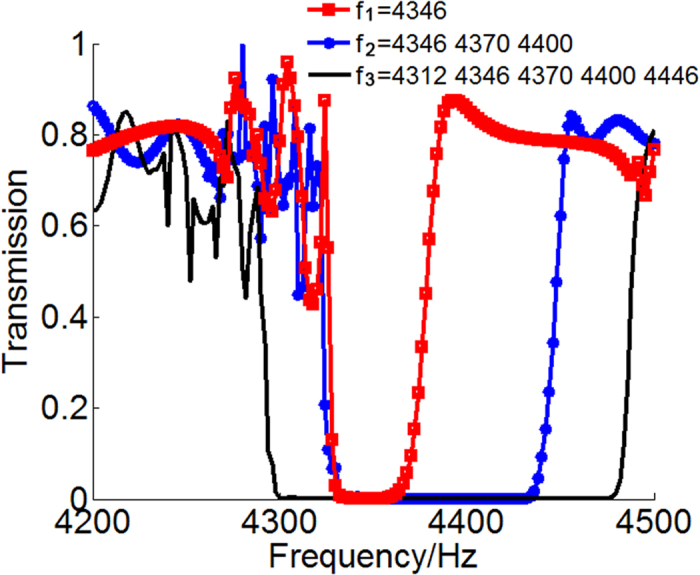
Three different hybrid structures with extendable working bandwidth. Simulation results of sound intensity transmissions for three different cases: *f*_1_ = 4346 Hz, *f*_2_ = 4346, 4370, 4400 Hz and *f*_3_ = 4312, 4346, 4370, 4400 and 4446 Hz.

Next we will discuss the extendibility of the working bandwidth of our designed sound insulator with thin shell and open configuration. The proposed structure has been proven both theoretically and experimentally effective to work at predesigned frequency, blocking the transmission of incident wave without sacrificing the continuity of background medium. This suggests the possibility of producing sound insulation in a more wide frequency range by cascading several metasurface unit cells with different eigen-frequencies. When more unit cells are added to extend the working bandwidth of the designed insulator, the above theoretical analysis requires that the phase gradient on each AM must follow the rule of *dϕ*/*dx* = −*k* where the value of *k* depends on the corresponding operating frequency. For simplifying the design and fabrication of the device, here we choose to fix the phase difference between adjacent units as a constant of −*π*/4 and simply change the spatial interval to adapt to the different working frequencies. We verify such a possibility via numerical simulation on the frequency dependence of transmission efficiency of hybrid structures composed of different numbers of components with eigen-frequencies chosen from 4312, 4346, 4370, 4400 and 4446 Hz respectively to cover the target frequency range with the fewest types of units. For a comparison, three particular cases are selected: *f*_1_ = 4346 Hz, *f*_2_ = 4346, 4370, 4400 Hz and *f*_3_ = 4312, 4346, 4370, 4400 and 4446 Hz. The corresponding numerical results are plotted in Fig. 5. It can be seen that the series connection of different parts of pipes, engineered to give rise to desired phase modulation around their respective eigen-frequencies, helps to effectively broaden the working bandwidth of the resulting device. This would be significant for various practical applications of sound insulators that may need to control broadband noises.

## Discussion

In conclusion, we have presented a mechanism for insulating sound transmission in a hollow pipe with subwavelength thickness. Through carefully designing the structure parameters, we can block propagating wave in the waveguide while leaving an open channel allowing other substances to pass. Instead of relying on the mismatched impedance or viscous dissipation used in the traditional designs of sound insulators with blocked path and bulky size, here we use the metasurface that has vastly decreased weight and thickness but is capable to provide phase gradient needed for manipulating the direction of incident wave. Furthermore, the dimension of the device in terms of the wavelength can still be further reduced by increasing the coiling ratio of the labyrinthine metastructure. Theoretical analysis, numerical simulations and experimental results have shown the effectiveness of our proposal. With the unique advantages of thin shell, light weight and easy fabrication, our designs should open new route to design of sound insulation with great application potentials in a variety of practical scenarios such as duct noise control in architectural fields and mechanical fields.

## Methods

### Numerical simulations

Throughout the paper, the numerical simulations are conducted by the finite element method based on commercial software COMSOL Multiphysics^TM^ 5.1. The background medium is air whose mass density and sound speed is *ρ*_0_ = 1.21 kg/m^3^ and *c*_0_ = 343 m/s, respectively. Mechanical parameters of ABS plastic are mass density *ρ*_*A*_ = 1180 kg/m^3^ and sound speed *c*_*A*_ = 2700 m/s, which are the parameters of the 3D-printed materials in the experiments. The viscous effect has been ignored in simulations, corresponding to the experimental situation, where the thickness of viscous boundary layer, approximating to 0.024 mm for lower frequency limitation, is about 62.5 times smaller than the space of *w*_2_ between the plates.

### Acoustic measurements

The measurement is performed in the anechoic chamber in order to eliminate the undesired reflected waves. A 1/4-inch microphone (Brüel&Kjær type-4961) is used for measuring the sound field in the scanned region. A loud speaker is placed 2 m away from the pipe to obtain a plane wave incidence, emitting sound wave of frequency from 4200 Hz to 4500 Hz with a step of 10 Hz. Sound absorbing foams are also set at the exit of experimental insulator.

## Additional Information

**How to cite this article:** Zhang, H.-L. *et al*. Sound Insulation in a Hollow Pipe with Subwavelength Thickness. *Sci. Rep.* **7**, 44106; doi: 10.1038/srep44106 (2017).

**Publisher's note:** Springer Nature remains neutral with regard to jurisdictional claims in published maps and institutional affiliations.
